# A Report on New Remedies

**Published:** 1864-10

**Authors:** Edward B. Stevens


### MISCELLANEOUS.

### A REPORT ON NEW REMEDIES.

[Read before the Ohio State Medical Society, White Sulphur Springs, June 21, 22, 1864.]

By Edward B. Stevens, M. D., Editor Cincinnati Lancet & Observer.

[CONCLUDED FROM SEPTEMBER NUMBER.]

"The cases in which Dr. De Ricci has employed phloridzine with most success have been certain forms of atonic dyspepsia occurring in delicate females, to whom it was impossible to administer either bark, quinine, or salicine in any shape, without bringing on serious nervous excitement. He has also found it extremely well adapted for the treatment of young children of delicate constitutional habit, or when recovering from the whooping cough, infantile fever or any other disease. The doses he has employed are five grains three or four times a day for adults, and proportionally small for children. In prescribing phloridzine it must be borne in mind that it is almost insoluble in cold water, but the addition of a very small quantity of ammonia instantly dissolves it, thus by adding to an eight ounce mixture containing a drachm of phloridzine a few drachms of aromatic spirit of ammonia the fluid which was previously milky becomes perfectly clear, and the addition of the aromatic spirit rather improves the mixture than otherwise. Dr. De Ricci relates the case of a young lady of a strumous constitution, suffering from chlorosis, in which the effects of phloridzine were manifestly favorably. The patient was unable to take iron in any shape, and both quinine and salicine equally disagreed with her; but phloridzine agreed perfectly well, and her constitution improved so much under its use, that she was subsequently able to take citrate of iron and strychnia in grain doses, which ultimately effected a perfect cure. Dr. De Ricci thus recapitulates the advantages of this drug:

"It is tolerated in cases where neither quinine, nor salicine, nor bark can be administered with impunity:

"It is particularly adapted to young children:

"It is not expensive—thus rendering us independent of the rapidly diminishing cinchona forests of South America."

*Ergot of Wheat a substitute for Ergot of Rye.*—Physicians who are in the habit of using the ergot of rye, have always experienced certain inconveniencies which tend to deteriorate its actual efficacy and render its action constantly uncertain; these are particularly—the amount of poisonous resin which is contained, and the action of time and damp in rendering it absolutely inert. These objections are sought to be avoided by the substitution of the ergot of *wheat* for the rye heretofore so well known. We find the following paragraph in the *New York Independent* for June 8th:

"The ergot of wheat is proposed by M. Leperdriel of Montpelier. It is much rarer than the ergot of rye, but can be found in sufficient quantity. Its color is much the same as that of rye, but differs in shape. Whilst the ergot of rye is fusiform, generally curved like the spur of a cock, and furrowed longitudinally with striæ of equal length, the ergot of wheat preserves the form of the grain which it replaces, is deeply cleft, and is often even divided into two, and sometimes into three at its upper extremity. It has the remarkable physical property of resisting decay, and hence of preserving for a length of time its medical virtues. It can thus be kept many years without undergoing any alteration. It moreover contains 15 per cent. less of the poisonous principle of ergots, and yield 20 per cent. more of the efficacious principle. Such are the reasons which lead Mr. Leperdriel to prefer ergot of wheat to that of rye.

*Caulophyllum Thalactroides as a parturient.*—In the series of articles to which we have already referred in the *London Lancet*, it is stated that the caulophyllum thalactroides which we believe belongs to the *cohosh* family; and therefore may probably resemble the cimicifuga racemosa, is a parturient of more decided reliable efficacy than the ergot. Its mode of administration and dose is not given, but we suppose should be given as an infusion—or what would be better—as a fluid extract, of which ʒss to ʒj would be a proper dose.

*Liquor Bismuthi.*—Most practitioners agree in opinion as to the special value of bismuth in painful affections of the stomach, however much they may differ as to the nature of the pathological conditions giving rise to these very common painful states of the organ. We have hitherto been confined to two preparations—the tris nitrate and carbonate- Both these are insoluble powders, bulky and inconvenient, inasmuch as a single dose cannot be made into one or two pills.

The *Lancet* for September, 1863, states that Mr. Schacht of Clifton, has succeeded in preparing a solution of bismuth, which is uniform in composition, stable, miscible with water or other fluids without precipitation, and is efficient in small doses. This solution is quite transparent, with a slight alkaline reaction, and although it contains only eight grains of oxide of bismuth in an ounce, a fluid drachm for a dose is found to be equivalent to a full dose—fifteen or twenty grains—of the insoluble tris nitrate.

A very excellent chemist and pharmaceutist—Mr. Wayne—at the store of Suire, Eckstein & Co, in Cincinnati, has been for some time preparing the liquor bismuth, and several physicians of that city have tested its efficacy and report very satisfactory results.

*McMunn's Elixir of Opium.*—For near a quarter of a century the secret nostrum known as McMunn's Elixir of Opium has been a favorite remedy with many physicians who have patronized it and praised it to the great delight of the proprietor, and the degradation of the profession.

The special, excellence originally claimed for McMunn's Elixir was that the opium was *denarcotized*, but it has long since been very well established that *narcotine* possesses no narcotic principle. It is at least harmless, if not a safe anti-periodic. Recent articles in the *Philadelphia Reporter* and the *New York Independent* give the entire *rationale* of the preparation, from which we learn in brief the following steps in the process:

1st—The opium is subjected to sulphuric ether, which is supposed to remove the narcotine, as also its peculiar noxious odor.

2d—A process of boiling follows to remove the sulphuric ether.

3d—A watery solution is made and the opium is macerated for six days, after which

4th—Alcohol is added in certain proportions, after standing undisturbed a few weeks it is the elixir, and is fit for use.

Reliable chemical analysis proves that the preparation thus made is for efficiency *only a solution of morphia* that the process leaves the morphia, narcine and extractive matters only depriving the opium of its pseudo morphia, cordeira, narcotina, thebana, meconine, fatty matter and resin. The narcine and extractive matters contained are so nearly inert that after the precipitation of the morphia, the liquid might be taken in doses of an ounce without injury. It seems then demonstrated that the so long vaunted Elixir of McMunn is nothing more than a solution of impure morphia.

*Saracenia Purpurea.*—Perhaps no new remedy has attracted more general professional interest and attention than the American pitcher plant, for the treatment of variola; and the importance of its claims will be sufficient apology for occuyying some unusual space in its notice. The saracenia purpurea, or American pitcher plant, grows abundantly in various parts of the United States, and first came into notice about four years ago, as the "Indian Remedy" for small pox, and was introduced to professional notice by British army surgeons on duty in Nova Scotia. They claim for it that it not only relieves, but actually *extinguishes* the disease; renders the variolons poison effete; that its special manifestation is first to encourage the appearance of the eruption, then to abort it, i. e. that very speedily the eruption dessiccates, and scales off without rendering the patient liable to pitting, or any of the terrible train of this loathsome disease in its usual progress. It was in addition claimed that the *saracenia* is a most reliable and efficient remedy for inveterate cutaneons affections as psora, lepra, etc.

As used by the Indians, and as introduced by Drs. Miles and Morris, the *decoction of the root* was alone recommended, the old original squaw claiming that the root alone possessed anti-variolous properties. Other writers however report the use of the entire plant indiscriminately; for instance Dr. McDowell, Act. Asst. Surgeon U. S. A., at Trenton, Mo., in an article in the *American Medical Times*, of September 5th, 1863, used the leaves as he was unable to procure the root, and administered the decoction of the leaves in the strength of 1½ ℥ to a quart of boiling water, a wine glassful of this strength being administered every 6 hours. He reports 43 cases treated with this remedy in the U. S. General Hospital, at Trenton, and the results fully or mainly confirming the claims originally set up by Miles and Morris; that is to say that the patients treated with saracenia had less secondary fever, the eruption speedily aborted, little or no pitting followed.

On the other hand, several very careful observers have reported their experience as having no appreciable result confirming the good effects of the remedy.

Dr. Noah C. Levings, of New York, reports his experience in several cases, in which he had "obtained the contused root of the sarrcenia purpuria direct from Maj. Lane, of Halifax, the putative father of the specific." In the observations of the group of cases put to this test, Dr. Levings called in Dr. Jacobi, a well known New York practitioner, and teacher, to watch the progress of the cases, so that we have every reason to regard the experiments as made carefully and without prejudice for or against the success of the remedy. His report is that there was no increase of urine, no flattening of the eruption, but that in every respect these cases passed consecutively through the regular and customary stages of variola; the remedy in no respect manifesting any appreciable effects upon the character or duration of the several cases.

Dr. Goyder reports in the *London Lancet*, a single case treated with the root infusion according to the directions of Dr. Miles: A child, aged 3 years, came under treatment October 28th—eruption already papular and tending to confluence; gave the saracenia in table spoonful doses; October 31—vesicles becoming pustular; November 1—the eruption wherever not abraded by the rubbing of the patient, are much flatter than usual, and he supposed the remedy was beginning to manifest its supposed virtues, especially as neutralizing the vitality of the pustule, and the variolous poison; that night however the patient died.

It has so happened that I have had an opportunity during the past year to test the remedy to some extent, as physician to the Cincinnati Pest House. 108 cases of small pox were under my care during the seven months following July 1st, 1863. Of these 108 cases, nearly all were subjected to the free use of the decoction of the *leaves* of the saracenia purpurea—not being able to procure the root—the decoction was prepared of the strength of 1½ ℥ of leaves to the quart of infusion, and was administered freely as a drink, from 6 to 8 ℥ of this infusion being given during the day. Some of these cases had measureably run their course previous to admission. Some were mild cases, essentially but simple varioloid; of course these were no test of the effect of the remedy. About 75 cases were fairly submitted to the influence of the remedy. In a few of the cases I thought there was an abridgment of the duration of the cases, and that the pustules dried up more speedily and scaled off more promptly than is usual. But in the great majority of these cases I saw no difference in the progress of the disease from that usual in cases of like malignancy. The mild cases run a mild and manageable course as is usual; the well marked and confluent cases run a course unabated in any respect from its usual virulence and completeness. The pustulation was as full, the heavy flakes of scales as large, and the condition of the patient in every way quite as offensive. In the well marked cases there was the same proportion of pitting, and in no case did I observe that the secretion of urine was affected either in character or quantity.

In the treatment of these 108 cases, the remedy was administered in every stage of the disease, administered freely and with a desire for its success. My conviction was that no more impression was made upon the disease than would be by any other herb tea. I am therefore inclined to accept as correct the conclusions of the committee on Intelligence of the New York County Medical Society. 1st. That the analyses already made of the plant do not give any active principle or element which would indicate any great medicinal potency. 2d. That the discoverers and advocates of the specific remedial power of the saracenia purpurea over variola have given too great credit to the *post hoc* circumstances, as being *propter hoc* influences. 3. That the reliable recorded experience thus far appears to preponderate against the medicinal efficiency of this plant in those forms of disease which do not generally recover under the administration of ordinary remedies."

*The Calabar Bean.*—No new remedy has perhaps attracted more interest of late than the calabar bean, especially amongst practitioners devoted largely or specially to eye surgery. We are indebted to Dr. Christison for bringing the peculiar properties of this drug to notice. In his personal experience he found that 12 grs. of the powdered bean produced serious and dangerous symptoms of poisoning, accompanied with remarkable contraction of the pupil. It is now found that a solution of the extract, or a tincture, if applied in small quantities to the eye produces this contraction of the pupil in a singularly marked degree. In fact in this respect its therapeutical action being exactly the reverse or the antagonist of the belladona; and if atropine be applied to one eye and a tincture of the calabar bean to the other, the two extremes of therapeutic effect are most remarkable. One of the most readily occurring uses of this remedy would suggest itself as counteracting the use of atropine for its usual purpose in eye surgery; but undoubtedly its application in eye surgery alone will prove much more extended than this, even though its effects as a peculiar poison should not render it available for other purposes.

*Per Manganate of Potash.*—Another new remedy is attracting some attention, not particularly as a new salt, but from the fact that new properties and applications of it are proposed. Dr. Samuel Jackson, of Philadelphia, has contributed for the *American Journal of Medical Science* for January, an article on the permanganate of potash, as a rapid developer of ozone in the human system; and hence as likely to become an important remedy in the treatment of low forms of disease, especially those forms of disease dependent on a depraved condition of the blood, or a condition of the blood faulty as to its oxygination; as for instance: erysipelas, hospital gangrene, typhus fever, and the like. As bearing somewhat on this remedy, we quote a paragraph cut from one of the papers of the day:

Ozone water is now used for drinking and the toilet. It is advertised in London in the following style: "Its use is attended by a sensation which has been aptly described as the 'perfume of purity.' Being perfectly inoxious and tasteless, a few drops make a most refreshing and invigorating addition to the tumbler of plain drinking or soda water, from which they remove all trace of soluble organic matter—a fact of infinite importance to the voyager or the invalid. When employed for the toilet, bath, etc., it removes from the mouth all impure and foreign tastes and odors, whether arising from natural or artificial causes, such as the practice of smoking, and counteracts the irritation and morbid effect of carious teeth. It purifies and softens the skin, and tends to promote a healthy state of the whole body, by removing all secretion, and restoring a wholesome condition."

Now Dr. Jackson states that this ozonized water of the English is a solution of the permanganate of potash and water in the proportions of 2 parts to 1000. In dyspeptic conditions of the stomach he found this simple ozonized water had a decidedly tonic effect in doses of a teaspoonful three or four times a day. As a local application to ulcers it stimulated to a process of healthy action and cicitrization. In hospital gangrene it was given internally and applied locally; internally it was used after the following formula: ℞—permanganate of potash ℥; acid sulph. gtt. xx; aqua font. Oij, which is about 2 grs. to the oz. Of this one teaspoonful was directed every three hours in a wine glassful of water. Its good effects when applied locally were almost immediate.

Acting upon the hints in this paper of Dr. Jackson's we learn that our fellow member, Dr. Dunlap of Springfield, has experienced most gratifying effects from the use of the permanganate of potash in the treatment of "spotted fever," as it appeared recently in and about that city. He gave it in the form just noted; and in the more malignant cases increasing the dose from $\frac{1}{8}$ to $\frac{1}{2}$ gr. frequently repeated; his theory being that in epidemic spotted fever we have a depraved condition of the blood resembling that of malignant scarlatina, or erysipelas.

Thus we might proceed, and still to considerable extent swell the matter of this report. We feel, however, that the patience of the Society has been sufficiently trespassed upon, and leave for future more careful gleaners to bring up the changing and improving progress which this department of medicine is making in its annual march.

